# Molecular Dynamics Simulation of Nanocellulose-Stabilized Pickering Emulsions

**DOI:** 10.3390/polym13040668

**Published:** 2021-02-23

**Authors:** Ka Kit Lee, Darren Yi Sern Low, Mei Ling Foo, Lih Jiun Yu, Thomas Shean Yaw Choong, Siah Ying Tang, Khang Wei Tan

**Affiliations:** 1 School of Energy and Chemical Engineering, Xiamen University Malaysia, Sepang 43900, Selangor Darul Ehsan, Malaysia; CME1609579@xmu.edu.my (K.K.L.); mlingfoo17@gmail.com (M.L.F.); 2 Chemical Engineering Discipline, School of Engineering, Monash University Malaysia, Bandar Sunway 47500, Selangor Darul Ehsan, Malaysia; darrenl333.dl@gmail.com; 3 Faculty of Engineering, Technology and Built Environment, Kuala Lumpur Campus (North Wing), UCSI University, Lot 12734, Jalan Choo Lip Kung, Taman Tayton View, Cheras 56000, Kuala Lumpur, Malaysia; yulj@ucsiuniversity.edu.my; 4 Department of Chemical and Environmental Engineering, Universiti Putra Malaysia, Seri Kembangan, Serdang 43400, Selangor, Malaysia; csthomas@upm.edu.my; 5 Advanced Engineering Platform, School of Engineering, Monash University Malaysia, Bandar Sunway 47500, Selangor Darul Ehsan, Malaysia; 6 Tropical Medicine and Biology Platform, School of Science, Monash University Malaysia, Bandar Sunway 47500, Selangor Darul Ehsan, Malaysia

**Keywords:** nanocellulose, molecular dynamics simulation, Pickering emulsions

## Abstract

While the economy is rapidly expanding in most emerging countries, issues coupled with a higher population has created foreseeable tension among food, water, and energy. It is crucial for more sustainable valorization of resources, for instance, nanocellulose, to address the core challenges in environmental sustainability. As the complexity of the system evolved, the timescale of project development has increased exponentially. However, research on the design and operation of integrated nanomaterials, along with energy supply, monitoring, and control infrastructure, has seriously lagged. The development cost of new materials can be significantly reduced by utilizing molecular simulation technology in the design of nanostructured materials. To realize its potential, nanocellulose, an amphiphilic biopolymer with the presence of rich -OH and -CH structural groups, was investigated via molecular dynamics simulation to reveal its full potential as Pickering emulsion stabilizer at the molecular level. This work has successfully quantified the Pickering stabilization mechanism profiles by nanocellulose, and the phenomenon could be visualized in three stages, namely the initial homogenous phase, rapid formation of micelles and coalescence, and lastly the thermodynamic equilibrium of the system. It was also observed that the high bead order was always coupled with a high volume of phase separation activities, through a coarse-grained model within 20,000 time steps. The outcome of this work would be helpful to provide an important perspective for the future design and development of nanocellulose-based emulsion products, which cater for food, cosmeceutical, and pharmaceutical industries.

## 1. Introduction

Food emulsions are common techniques employed in the food and beverages industry for more than 50 years. The coupling of food-grade surfactants (e.g., protein and amphiphilic polysaccharide biopolymers) to contain food items and oils (e.g., omega-3 fatty acids from fish oil) has enabled mankind to access numerous health benefits [1]. Particle-stabilized emulsions, or commonly referred to as Pickering emulsions, have recently emerged to be an alternative due to their better stability against coalescence and flocculation [2,3]. The Pickering stabilization mechanism is fundamentally different from conventional emulsions [4]. It is stabilized by the irreversible adsorption of solid particles on the oil-water interface, forming a dense layer of coating to prevent the aggregation of droplets. In addition, a Pickering emulsion could be more biocompatible if the substituting solid particles are generally regarded as safe by the Food and Drug Administration (FDA) [5].

Nanocellulose (NC) extracted from renewable resources has opened new opportunities for future Pickering emulsion development owing to its intrinsic biodegradability and renewability. Through chemical modifications [6] or grafting techniques with fatty acids, the surface wettability of NC in organic solvents [7,8] can be modified, thereby enhancing the emulsion stability. In addition, NC can be rendered with hydrophobic behaviors to bend the oil-water interface towards the water phase for the formation of reverse Pickering emulsions (or so-called water-in-oil Pickering emulsions) [9]. Albeit of the active community to improve the role of NC in the emulsion preparation, the effect of increasing the oily disperse phase or NC on phase invert activities is rarely investigated. For instance, the inversion of NC-stabilized Pickering emulsions from oil-in-water to water-in-oil droplets with the increasing oily phase (oil content) in water continuous phase remains unclear.

Cellulose is a linear chain homopolymer that consists of (1→4)-β-d-glucose monomers. It is synthesized by the cellulose synthase complex and exists abundantly in plant cell walls [10,11]. The hydroxyl groups of cellulose are equatorially placed on its glucopyranose rings, corresponding to the crystal plane $\left(1\bar{1}0\right)$ and a high hydrophilicity shall be expected for this surface [12]. In contrast, the (110) surface is hydrophobic, and the surface energy would be much lower than that of the $\left(1\bar{1}0\right)$ surface. Due to the hydrophobic effect, the cellulose chains may stack via the hydrophobic interactions and form a sheet-like structure where hydrophobic chain faces meet between the layers [13]. Over the decades, numerous works have been focused on the preparation of NC-stabilized emulsion using the NC of different origins or surface properties. The studies of emulsion formation usually involve a detailed investigation in terms of the relation between the particle concentration and the resulting emulsion properties, the mechanism of emulsion stabilization, and the stability of emulsion upon storage. The process can easily take up to months for the sample preparation, analysis, and repetition of experiments. Moreover, the transient changes within the emulsion system may be an obstacle to the understanding of Pickering stabilization.

Molecular dynamics simulation (MDS) has emerged as an effective tool in the prediction of the physical movement of atoms and molecules at the nanometric scale. For instance, the improved mechanical properties of a PI/SiO_2_ nanocomposite as compared to pure PI could be well justified via MDS before being developed into reinforced materials [14]. However, the application of MDS in NC-stabilized emulsion systems is rarely reported. MDS in a colloidal system could be useful to fill up the gap left from physical laboratory work, particularly to provide insights into the interfacial stability, fluidity properties, and thermodynamic quantities [15,16]. It is particularly useful when surface modification was involved to improve NCC properties such as wettability [3,17]. For instance, the extraction of NC from oil palm leaves can be facilitated via MDS to investigate the role of fungal lignocellulosic enzymes, i.e., β-glucosidase and xylanase of *Trichoderma asperellum* UC1 on the NC isolation. The details of the molecular docking of the enzyme-substrate complex would be helpful to optimize the overall economics of processing lignocellulosic materials in the production of NC [18]. After that, the surface chemistry of NC could be further altered (with the aid of MDS) to improve their dispersibility in on-polar polymers [19]. Coarse-grained modelling was greatly utilized in biomolecular systems that involve mainly carbohydrates, where the number of atoms or molecules were reduced into a single bead to interact with other pre-defined beads in a dynamic system. For example, the self-assembly of oil-in-water emulsion due to the strong hydrophobicity effect could be quantitatively observed through active interfacial activities [20,21]. To simplify the study, this work attempted to investigate the role of NC in emulsion formation and its phase inversion behavior, by assuming the NC degree of polymerization to be 20 in the sketching of (1→4)-β-d-glucose in a syndiotactic polymer arrangement [22,23], with a hydrocarbon polymer, i.e., poly-1-butene (PB) to be the oil phase with a polymerization degree of 7 [24]. The simulation outcome could significantly reduce the number of trials during the formulation development and could be a referencing material for the experimentally prepared Pickering emulsion. It was speculated that the amphiphilic properties of the NC would lead to self-assembly, while the hydrophobic effects of both the oily phase and NC would push them forward to aggregate, resulting in a noticeable change in morphology. An analysis of the thermodynamics and bead orders would thereafter be reviewed to provide more insights into the transient dynamics of the colloidal system. Furthermore, employing MDS could facilitate the process of emulsion studies, particularly it could minimize the cost, time, as well as the effort to determine the emulsion formulation for the initiation of experiments.

## 2. Materials and Methods

### 2.1. Molecular Dynamics Simulation

**Figure 1:**
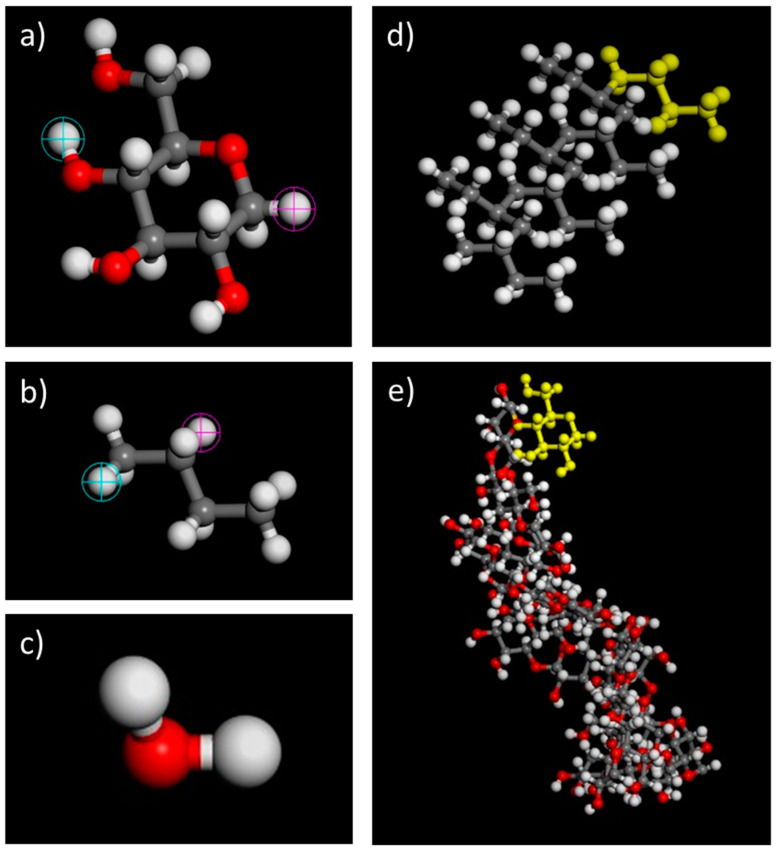
Molecular structure of (**a**) (1→4)-β-d-glucose, (**b**) 1-butene, (**c**) water, (**d**) poly-1-butene (PB) (degree of polymerization = 7), and (**e**) nanocellulose (NC) (degree of polymerization = 20). The blue and purple circles in (**a**,**b**) were identified as the head and tail where polymerization occurs. Yellow structures in (**d**,**e**) represent the monomer of 1-butene and glucose, respectively.

**Figure 2:**
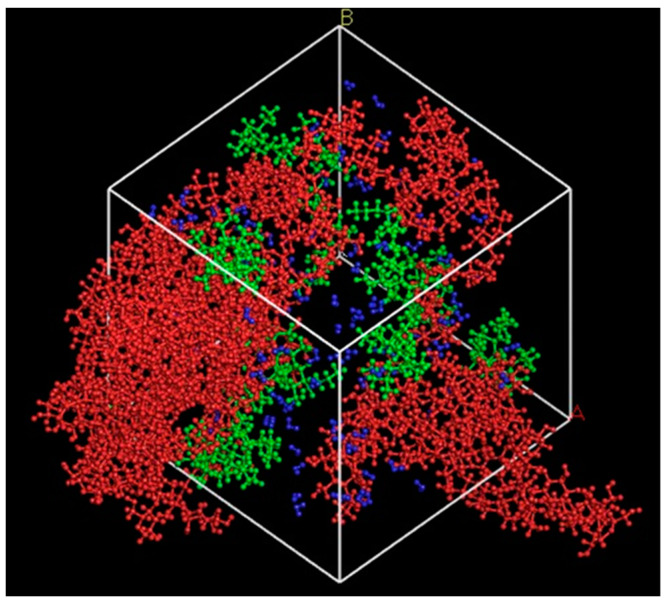
Basic dynamics of NC (red), PB (green), and water (blue) in a cubic lattice of 68.7 Å with molecular loading of 10, 10, and 100, respectively.

Material Studios 8.0 (BIOVIA, United States) was employed to sketch and compute the molecular movement in the mesoscopic dynamics (MesoDyn) coarse-grained simulation. The molecule of (1→4)-β-d-glucose and PB were imported from the system library as the modelling molecule structure of NC and oil, respectively, while the water molecule was sketched manually, as shown in Figure 1. A geometry optimization procedure via Forcite was performed to ensure that the modelling molecule structures were at the most stable state (energy minimization) before initiating the simulation [25]. The basic dynamics of the above-mentioned molecules could be obtained in an amorphous cell [26], as depicted in Figure 2.

### 2.2. MesoDyn Coarse-Grained Calculation

The MesoDyn calculation mainly relied on two inputs, namely, Gaussian Chain topologies and Flory-Huggins expression [27]. Chain topology is a measurement of the degree of coarseness. In general, each molecule would be separated into beads of equivalent chains, so that the number of items would be approximated to a value that was accurate enough to define the dynamic interactions among the molecules. There were several approaches to determine the Gaussian chain of a polymer [28], however, it can be generally defined as follows:

(1) $$N_{\mathrm{meso}}\;=\;\frac{M_{p}}{C_{n}M_{m}}$$

where N_meso_ is the number of molecules per bead, M_p_ is the molecular mass of polymer per bead, M_m_ is the molecular mass of monomer per bead, and C_n_ is the characteristic ratio unique to the polymer. All the basic properties were referred from the Synthia module [29,30]. To employ (1), the value of each term of individual molecules is summarized in Table 1.

The Flory-Huggins expression, X_ij_ derived from Van Krevelen solubility relates to the interaction energies of molecules, ε_ij_ among the different beads and can be expressed as (2) [31].

(2) $$X_{\mathrm{ij}}\;=\;\frac{\beta }{2v}\left(\varepsilon_{\mathrm{ij}}{\;+\;\varepsilon }_{\mathrm{ji}}{\;+\;\varepsilon }_{\mathrm{ii}}-\varepsilon_{\mathrm{jj}}\right)$$

where β = 1/kT, and v is the average bead volume. With MesoDyn, the X_ij_ can be simplified into (3) based on the Van Krevelen solubility parameter, δ [31].

(3) $$X_{\mathrm{ij}}=\frac{V_{\mathrm{ref}}\;{\left(\delta_{i}-\delta_{j}\right)}^{2}}{\mathrm{RT}}$$

where V_ref_ is the average of two bead monomers, R is the gas constant 8.314 J/mol.K, and T is the absolute temperature, 298 K [31]. However, for a system containing an aqueous phase (usually in a pluronic mixture), the value of X could be varied from the literature [27,32]. This could be due to the presence of hydrogen bonding in aqueous solutions. As such, the value of X could be revised using (4).

(4) $$X_{\mathrm{ij}\;}{=\;\theta }^{-2}\left[\ln\left(\frac{p}{p^{0}}\right)-\ln\left(1\;-\;\theta \right)-\left(1-\frac{1}{N}\right)\theta \right]$$

where θ is the polymer volume fraction, p is the vapor pressure of the polymer mixture solution, and p^0^ is the vapor pressure of the pure solvent [33]. A summary of the derivatives is shown in Table 2.

The coarse-grained modelling was employed for the mesoscale interaction among the studied molecules. It replaced the All-Atom (AA) modelling by coursing or averaging the atomic details of a molecule, thereby facilitating the study of mesoscale length scales [28,32]. The MesoDyn coarse-grained modelling was devised based on the time-dependent mean-field density-functional theory. The free energy F of an inhomogeneous liquid would be used as a function to the local density approximation ρ [34]. In the model of Gaussian chain, various pre-defined beads (e.g., i, j, etc.) were denoted to demonstrate the intramolecular dynamics, and can be described using the functional Langevin Equations (5) and (6) that take the component densities and the noise of a system into account.

(5) $$\frac{\partial \rho_{i}}{\partial t}{\;=\;\mathrm{Mv}}_{j}\nabla \rho_{i}\rho_{j}\nabla \left[\mu_{i\;}-\mu_{j}\right]\;+\;\eta$$

(6) $$\frac{\partial \rho_{j}}{\partial t}{\;=\;\mathrm{Mv}}_{j}\nabla \rho_{i}\rho_{j}\nabla \left[\mu_{j}\;-{\;\mu }_{i}\right]\;+\;\eta$$

where Mv_j_ is the mobility of a bead determined by Mv_j_∇ρ_i_ρ_j_ (kinetic term), η (blank Gaussian noise), and μ (diffusion coefficient). The above information is essential for the creation of density fields of a heterogenous system, which is an important factor in observing the phase separation of a colloidal system represented by different beads in the modelling system.

To evaluate NC as a particle stabilizer in an oil-water mixture, the molecule models were monitored in a 32 × 32 × 32 nm simulated cubic cell within 20,000 time steps. The concentration of NC, PB, and water was altered to study the formation of oil-in-water (O/W) emulsion, water-in-oil (W/O) emulsion, as well as the oil-water bi-continuous structure. The concentration of NC and PB was added with respect to the amount of water. To observe the formation of O/W emulsion, the ratio of NC to water was doubled as compared to the ratio of PB to water. Similarly, the concentration of PB would be higher than NC for the formation of bi-continuous structure. Lastly, the formation of the bi-continuous structure was studied by gradually increasing the ratio of both NC and PB to water from 1:10 to 9:10.

## 3. Results and Discussion

### 3.1. Formation of Pickering Emulsion with an Excess Supply of Nanocellulose

**Figure 3:**
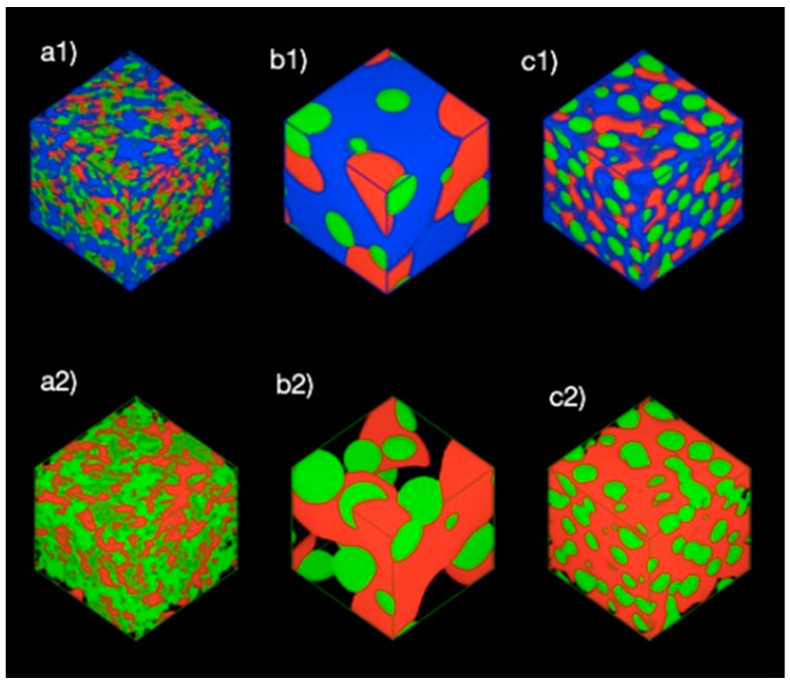
Phase morphologies of the systems in at the final frame of 20,000 time steps. The ratio of NC (red), PB (green), and water (blue) are (**a1**) 0.1/0.05/1, (**b1**) 0.4/0.2/1, and (**c1**) 0.8/0.4/1, respectively. The visualization of water was excluded from (**a2**,**b2**,**c2**) for clearer observation.

Figure 3 shows the phase morphology of the mixture at three different concentrations. It showed no noticeable spheres or micelles that were formed when the ratio of NC and PB were fixed at 0.1 and 0.05, respectively (Figure 3a). When the ratio of NC and PB was increased to 0.4 and 0.2, respectively, the NC and PB slowly assembled into O/W emulsions (Figure 3b). The phenomenon continues as the simulation proceeded, leading to increased amount of emulsion droplets with the smaller droplet size (Figure 3c). In general, the oil phase would be stabilized by the adsorption of smaller particles (i.e., NC in this study) at the oil-water interface, where the properties of NC would determine the emulsifying behavior. The particle attachment could lower the interfacial tension of oil and water, creating more surface area for the formation of smaller Pickering emulsion droplets [35]. It has been suggested that the -CH groups on the glucose-pyranose rings of NC exhibit a hydrophobic behavior, while another part of its molecular structure contains hydrophilic hydroxyl and methyl hydroxyl groups to interact with the water phase. The hydrophobic PB first self-aggregated into PB beads, forming a temporary droplet until it contacts with the NC molecules to seek “shelter” in a more stabilized form of Pickering emulsion.

#### 3.1.1. Thermodynamic Properties with Increasing Nanocellulose Concentration

**Figure 4:**
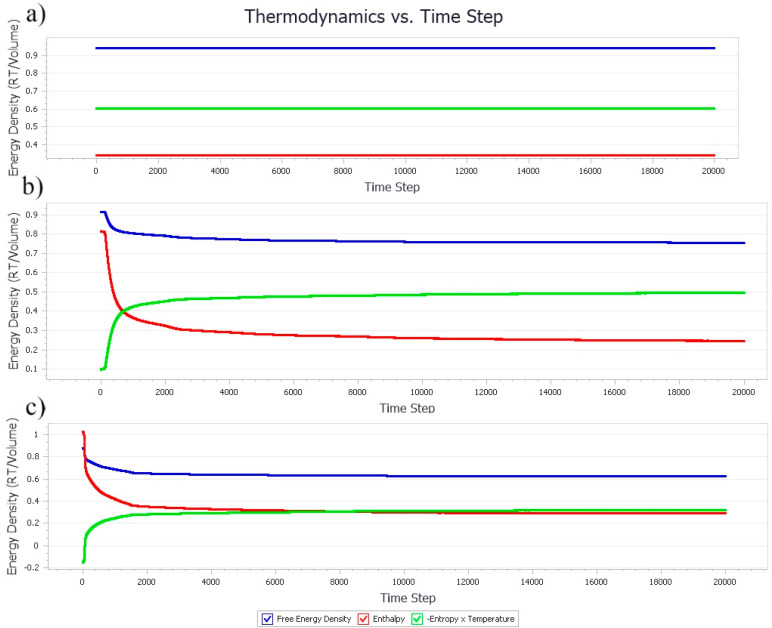
Thermodynamic density of emulsion systems with an increased concentration of NC. The ratio of NC, PB, and water were fixed at (**a**) 0.1/0.05/1, (**b**) 0.4/0.2/1, and (**c**) 0.8/0.4/1, respectively.

The formation of microemulsions is where the droplets or micelles would form spontaneously upon emulsification and remain thermodynamically stable [36]. It is to seek the energy balance for the entropic effect of due to the stabilization of oil-water surfaces [31]. A low concentration of amphiphilic polymer, such as NC in water as the continuous phase in the system tended to have random movements, indicating a homogenous phase morphology (Figure 4a). As the concentration of both NC and PB increases, the hydrophobic effect becomes dominant. The NC would then adsorb onto the oil-water interfaces, forming droplets to lower the free energy in the system. Referring to Figure 4b,c, the Gibbs free energy decreased for lower spontaneity of the system through the creation of more oil-water surfaces. In general, the changes in energy was first observed between 50 to 100 time steps, where the system was homogenous before the gradual take-over of polymer hydrophobic effects, as seen from the steep slope around 100 to 200 time steps. The formation of emulsion droplet was initiated, until the slope plateaued, and eventually stabilized after approximately 800 time steps.

#### 3.1.2. Effect of Nanocellulose Concentration on the Bead Order

**Figure 5:**
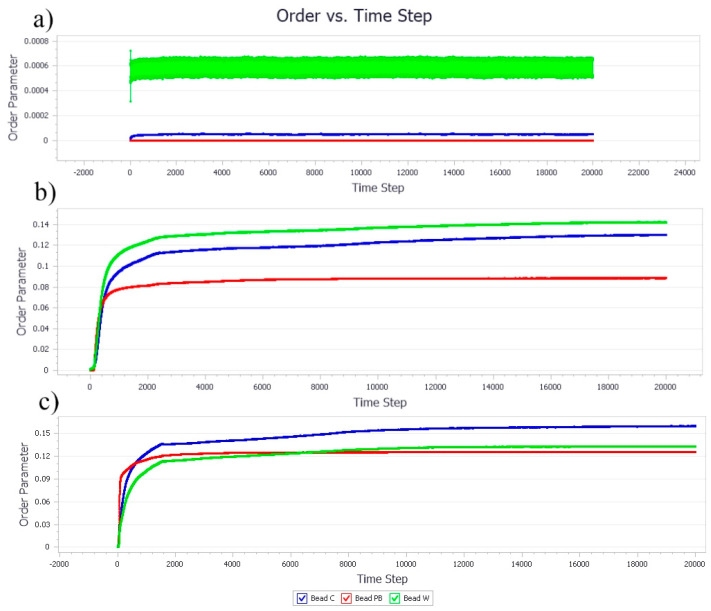
The evolution of NC, PB, and W bead order parameters as the concentration of NC increases. The ratio of NC, PB, and water are (**a**) 0.1/0.05/1, (**b**) 0.4/0.2/1, and (**c**) 0.8/0.4/1, respectively.

The bead orders of samples at different concentrations of NC and PB indicating the phase condition and sequence among the beads in the simulation models are presented in Figure 5. In general, the higher the bead order, the more stable the system. At a low concentration of particle stabilizer (i.e., NC ratio of 0.1), the bead order showed a linear trend for all the bead models and was close to zero throughout the studied time step of 20,000 (Figure 5a). This suggested that all three types of molecules were well dispersed in the system and no stable droplets were formed due to insufficient particles for Pickering stabilization. The bead orders in Figure 5b were exponentially increased with a higher concentration of NC and gradually stabilized at 200 time steps, suggesting the rapid formation of Pickering emulsions owing to the particle availability for droplet formation and stabilization. A further increase in the concentration of NC could accelerate the formation of droplets, leading to more aggressive formation and destruction of NC and PB aggregates, as indicated by the overlapping lines and relatively higher bead orders, shown in Figure 5c as compared to Figure 5b [31].

#### 3.1.3. Density Map with Excess Supply of Nanocellulose

**Figure 6:**
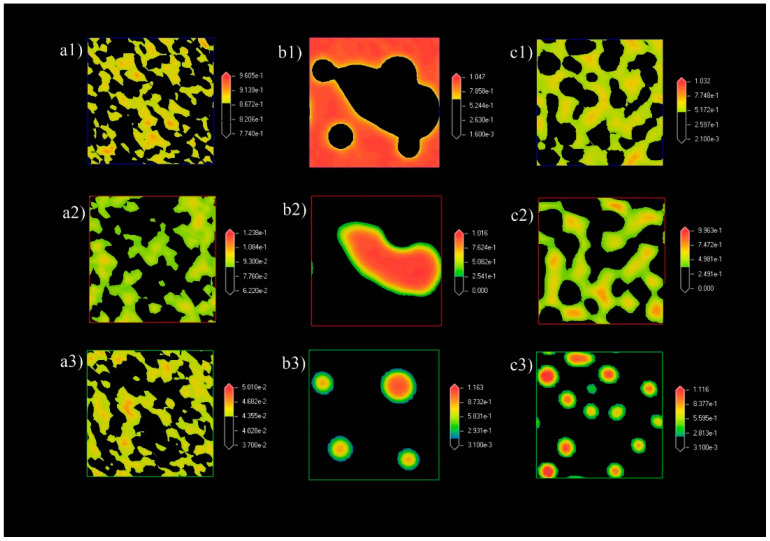
Density maps of (**a1**–**c1**) water, (**a2**–**c2**) NC, and (**a3**–**c3**) PB. The ratio of NC, PB, and water in the system were (**a1**–**a3**) 0.1/0.05/1, (**b1**–**b3**) 0.4/0.2/1, and (**c1**–**c3**) 0.8/0.4/1, respectively. All density maps were obtained at the final frame of 20,000 time steps.

Figure 6 shows the density maps of the system at different concentrations of NC and PB. The system presents a mixture of NC, PB, and water from Figure 6(a1–a3), where no emulsion droplets were observed from the density map. This could be ascribed to the low concentration of NC (0.1 by ratio) in the bulk water phase. An increase in the concentration of NC and PB to 0.4 and 0.2 by ratio, respectively has led to the formation of hydrophobic cores, where PB was bounded by NC (Figure 6(b1–b3)), which eventually resulted in Pickering emulsion droplets at the end of 20,000 time steps. In addition, it was also observed that increasing the concentrations of NC and PB further could create more oil-water surfaces for the formation of emulsion droplets with a smaller diameter, as shown in Figure 6(c1–c3). This observation is in agreement with the findings of Mortensen and Pedersen [37].

### 3.2. Formation of Pickering Emulsion with Excess Poly-1-Butene

**Figure 7:**
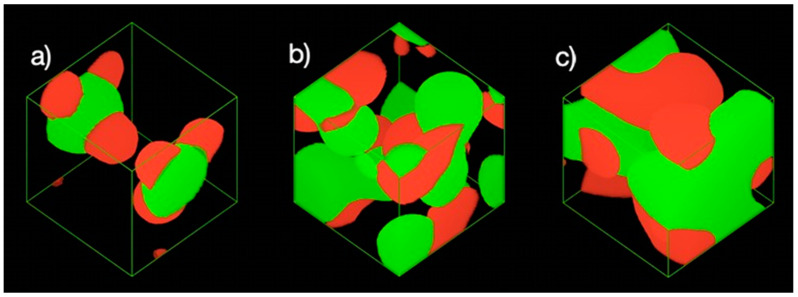
Phase morphologies of the systems at the final frame of 20,000 time steps. The ratio of NC (red), PB (green) and water are (**a**) 0.05/0.1/1, (**b**) 0.2/0.4/1, and (**c**) 0.4/0.8/1, respectively. Water was excluded for better observation.

The phase morphology of the systems at different concentrations of NC and PB in at the final frame after 20,000 time steps is presented in Figure 7a,c. PB droplets with NC aggregates adsorbed on its surface were observed in Figure 7a. This could be attributed to the hydrophobic C-H bonds located on the axial position of the glucopyranose ring, which could direct its hydrophobic planes to the oil phase, i.e., PB [38]. The droplets were inherently the O/W emulsion. However, the droplets coalesced rapidly due to the low concentration of NC relative to the amount of PB in the system. The droplets tended to arrange into a larger aggregate when the ratio of NC and PB was increased to 0.2 and 0.4, respectively, as shown in Figure 7b. Owing to the excess amount of PB as compared to the fraction of NC in the system, the low coverage of NC onto PB droplets eventually led to the large aggregates at the end of the time steps. The NC tended to have the higher affinity towards the adjacent NC due to the presence of hydrogen bonding between the hydroxyl groups of NC. Due to the hydrophilicity of NC and hydrophobicity of PB, the PB droplet clusters were segregated by the NC aggregates. However, no oil-water layers were observed up to 20,000 time steps. A further increment of the PB ratio to 0.8 with the NC ratio of 0.4 has caused the aggregation to deteriorate, as captured in Figure 7c. The PB aggregate clusters were merged into be an unstable mixture and mainly distributed at the upper part of the cubic model. We speculated that the gravitational effect could eventually lead to the separation of the oil layer over a prolonged period.

#### 3.2.1. Thermodynamic Properties with Increasing Poly-1-Butene Concentration

**Figure 8:**
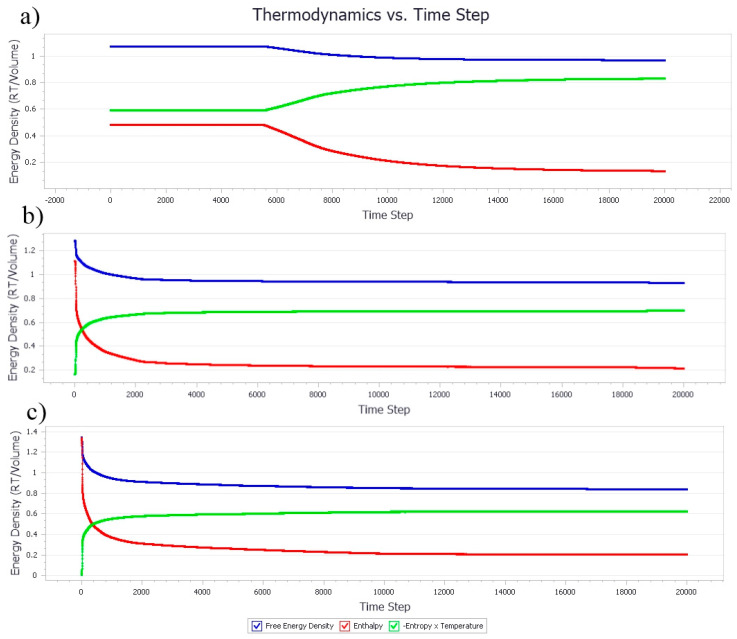
Thermodynamic density of emulsion systems with increased concentration of PB. The ratio of NC, PB, and water were fixed (**a**) 0.05/0.1/1, (**b**) 0.2/0.4/1, and (**c**) 0.4/0.8/1, respectively.

The free energy in the system containing the ratio 0.05 of NC and 0.1 of PB was homogenous until the time steps approached 5500 (Figure 8a), which could be attributed to the increase in the size and amount of PB aggregates. The hydrophobic effect between the PB aggregates and the attachment of the NC hydrophobic plane to the PB molecules could cause a change of entropy in the system and reduced the free energy of the system. The rapid coalescence of droplets continued and eventually reached a plateau after 10,000 time steps. The system was inferred to have reached its equilibrium state where the droplets attained a minimum coverage with the available NC. Increasing the ratio of PB in the system accelerated the reduction in the free energy and attained the plateau state rapidly, as shown in Figure 8b,c. The destabilization had led the PB aggregates to propagate into larger clusters at approximately 1500 time steps. The adsorption of NC onto PB via hydrophobic planes and the aggregation of NC due to hydrogen bonding have caused the system to remain static until the end of the simulation period.

#### 3.2.2. Effect of Poly-1-Butene Concentration on Bead Order

**Figure 9:**
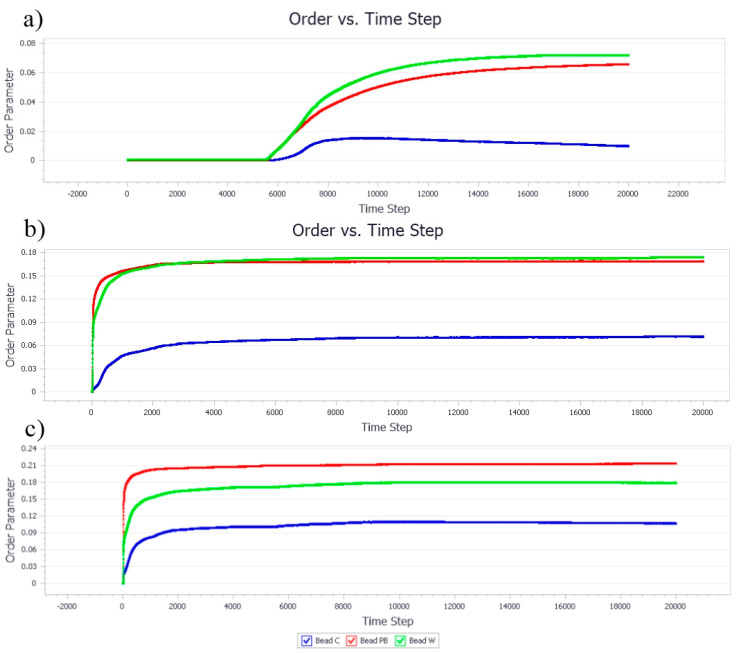
The evolution of NC, PB, and W bead order parameters as the concentration of PB increased. The ratio among NC, PB, and water were fixed (**a**) 0.05/0.1/1, (**b**) 0.2/0.4/1, and (**c**) 0.4/0.8/1, respectively.

At 0.1 of PB ratio, it was aligned with the thermodynamic profile that the phase separation had become more aggressive after 6000 time steps, as illustrated in Figure 9a. Likewise, the bead orders for PB at 0.4 and 0.8 ratio (Figure 9a,b, respectively) was recorded in a high order since the beginning, reflecting the thermodynamic profile of aggressive coalescence activities [39]. Both bead orders of NC and PB were relatively higher than the beads of water, suggesting the phase separation due to the hydrophobic effects of the beads trying to avoid contact with water molecules by “grouping” themselves into larger aggregates [40].

#### 3.2.3. Density Map with Excess Supply of Poly-1-Butene

**Figure 10:**
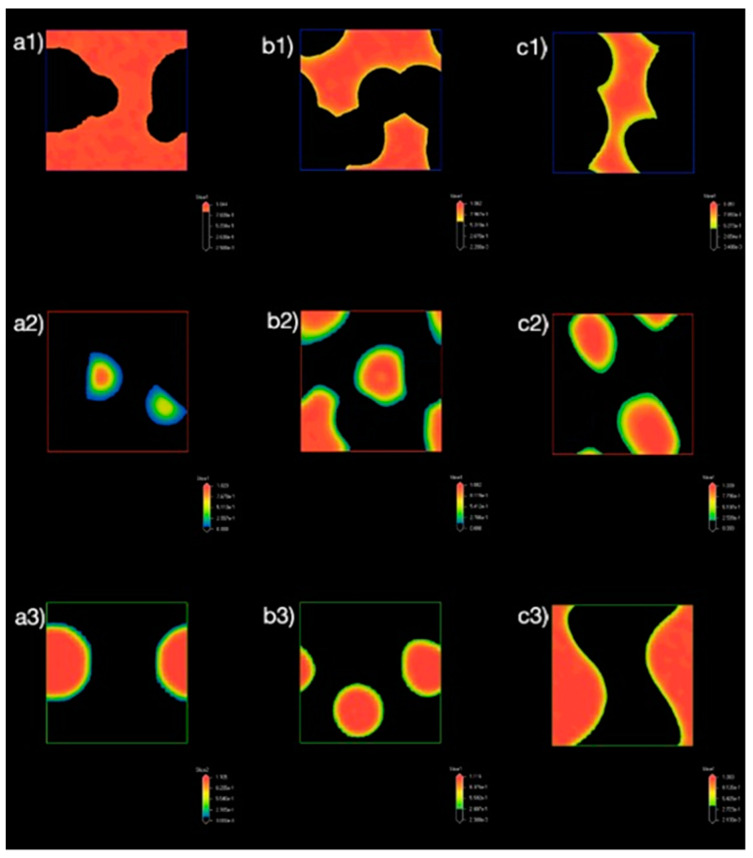
Density maps of (**a1**–**c1**) water, (**a2**–**c2**) NC, and (**a3**–**c3**) PB. The ratio of NC, PB, and water in the system were (**a1**–**a3**) 0.05/0.1/1, (**b1**–**b3**) 0.2/0.4/1, and (**c1**–**c3**) 0.4/0.8/1, respectively. All density maps were obtained at the final frame of 20,000 time steps.

The density maps of the systems with the increasing ratio of PB at the 20,000 time steps is presented in Figure 10. Comparing Figure 10a,b, the NC molecules distributed in the form of aggregation, where the NC aggregates partially adsorbed onto the PB droplets (Figure 10c). This observation is different when compared to systems simulated with excess NC (Figure 6), which could be ascribed to the nature of the properties and interactions between the NC and PB. As seen in Figure 6(b2,b3), single PB droplet was attached to multiple edges of NC aggregates, where one NC aggregate could adsorb on two adjacent droplets. This indicated that the adsorption of NC onto oil droplets, (i.e., PB) was achieved through the hydrophobic plane of NC when the oil fraction was in excess. In addition, droplets observed in Figure 6(c3) were found to be smaller compared to the droplet observed in Figure 6(b3), which could be contributed by the higher concentration of NC in the system and the adsorption mechanisms of NC. When the ratio of PB was further increased to 0.8 (Figure 10(c1–c3)), the larger PB droplets coalesced to reduce the oil-water interface, leading to the formation of oil-water bi-continuous structures [41].

### 3.3. Effect of Equal Ratio of NC and PB to the System

**Figure 11:**
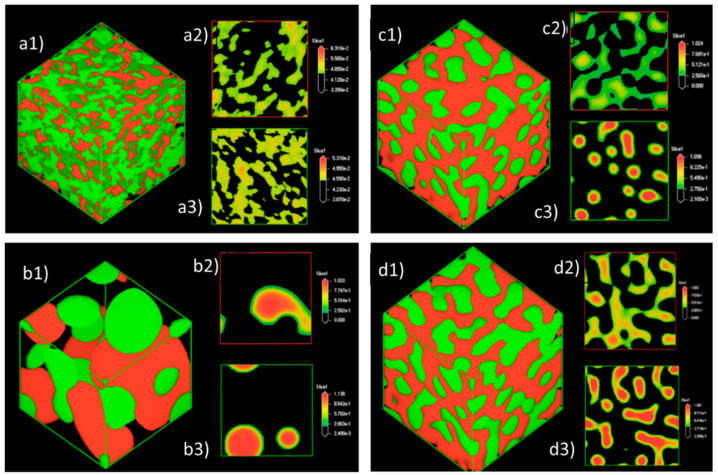
The phase morphology (red: NC; green: PB) in which the ratio of NC, PB, and water was fixed at (**a**) 0.05/0.05/1, (**b**) 0.3/0.3/1, (**c**) 0.5/0.5/1, and (**d**) 0.9/0.9/1, respectively; (**a2**–**d2**) the density maps of NC; (**a3**–**d3**) the density slides of PB. The results were obtained at the final frame of 20,000 time steps.

The cubic models with equal ratios of NC and PB were also simulated to investigate the effect of particle concentration and oil fraction to the emulsion stability. At an equal ratio of 0.05, the PB and NC were found to be homogeneously distributed in the system, as demonstrated by the phase morphology and density maps in Figure 11. Neither visible droplets nor phase separation was observed from the cubic system, which could possibly be due to the relatively diluted state of PB and NC presented in the bulk water phase (the fraction of water is approximately 20-fold higher than the amount of NC and PB).

The emulsion droplets were clearly observable in Figure 11b, when the ratio of PB/NC was increased to 0.3. The PB molecules tended to form spherical droplets in order to lower the free energy of the system with the available NC particles [34]. The spherical droplets slowly coalesced and merged into larger droplets until the system attained a state of equilibrium. It is worth noting that when the ratio of PB/NC was further increased to 0.5, smaller emulsion droplets were observed, as depicted in Figure 11c. As observed in Figure 11(c2), the NC distributed more evenly compared to Figure 11(b2). Some of the larger droplets were irregularly shaped and identified as the droplet clusters that were formed due to the coalescence of several droplets. At a PB/NC ratio of 0.9, the system developed into a bi-continuous-like structure of NC-PB mixture, as presented in Figure 11d. The droplets merged into the larger droplet clusters with NC aggregates dispersed within the system. Owing to the high fraction of PB, the movement of droplets could be inhibited by high viscosity and strong hydrophobic effects.

#### Effect of Equal Ratio of NC/PB to Free Energy Densities in the System

**Figure 12:**
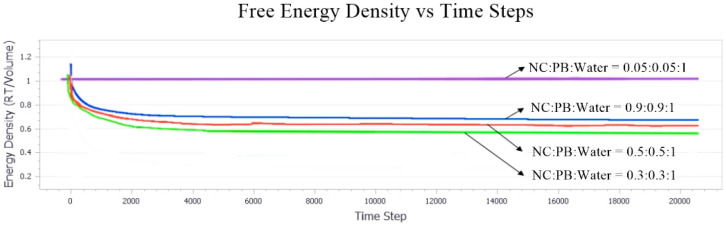
Free energy densities of the systems simulated with an equal ratio of NC and PB. The ratio of NC/PB (from top to bottom) was 0.05, 0.9, 0.5, and 0.3, respectively.

The free energy densities of the systems at different ratios were compared in Figure 12. At the equal ratio of 0.05, the simulation suggested no free energy change, which could be attributed to the relatively homogenous phase of the system where droplet formation or phase separation was negligible and insignificant. As aforementioned, the free energy change could be employed to identify the droplet formation within the system. A higher ratio of both NC and PB has induced the system instability, leading to an increase in system entropy, and the reduce in free energy of the system. As shown in Figure 12, the total free energy of these systems reduced over time because of the greater energy loss from enthalpy, which indicated the binding activity of NC and PB. The reduction in total free energy was 0.3 > 0.5 > 0.9, which suggested that a system that consists of NC/PB ratio of 0.3 is relatively favorable to the formation of stable emulsions. As the ratio of NC/PB increases, the higher fraction of PB contributed significantly to the movement and interaction of emulsion droplets in the system. Thus, a higher concentration of NC and a more intense emulsification process is usually required to create more surfaces for an effective droplet stabilization. This finding agrees with the phase morphology results simulated with the NC/PB ratio of 0.9, where the closely packed droplet clusters were coalesced and separated continuously, forming a highly unstable bi-continuous-like structure and often leading to phase separation at the end.

## 4. Conclusions

This study successfully demonstrated the phenomenon of Pickering stabilization using NC as a particle stabilizer in the PB and water mixture. In addition, the simulation successfully quantified the expected outcome, from the formation of Pickering emulsions to the bi-continuous structure. The self-assembly of a stable PB Pickering emulsion was often observed when NC was present in excess. It was also found that a thermodynamic highly unstable bi-continuous structure was formed when the concentration of both NC and PB approaches the concentration of water in the system. The findings of this study confirmed that molecular dynamics simulation is a useful tool in providing preliminary outcomes of a colloidal-stabilized emulsion system, particularly from the aspects of dynamic behavior with quantified profiles from a thermodynamic perspective. The current work could be greatly beneficial to future, extended studies in understanding preliminary Pickering stabilization phenomena through different types of biopolymeric particles.

## Figures and Tables

**Table 1:** Chain topology of each molecule bead. Water was assumed as one molecule per bead.

Molecule (Bead)	M_p_ (g/mol)	M_m_ (g/mol)	C_n_	N_meso_	Chain Topology
Nanocellulose (NC)	3244.84	164.157	4.37854927	5	C5
Poly-1-butene	394.772	58.124	7.32595465	1	PB1
Water (W)	-	18.015	-	1	W1

**Table 2:** Flory-Huggins parameter, X_ij_ between the beads.

Beads	C	PB	W
C	0	-	-
PB	12.2418032	0	-
W	3.8780612	13.1866039	0
			RT = 2477.572 J/mol

## Data Availability

Not applicable.
